# The Eighth Annual Meeting of the Western Dental Society

**Published:** 1864-12

**Authors:** 


### Proceedings of Societies.

### THE EIGHTH ANNUAL MEETING OF THE WESTERN DENTAL SOCIETY.

The Eighth Annual Meeting of the Western Dental Society was held at the "Tremont House," in Chicago, on the 23d day of July, 1864.

The meeting was called to order by the President, Dr. H. E. Peebles, of St. Louis, when the proceedings of the last Annual Meeting were read and approved. Drs. W. N. Morrison and W. H. Eames of St. Louis, and Drs. L. P. Haskell, John C. Fuller and J. Ward Ellis, of Chicago, having been duly proposed, became members of the Society.

Dr. I. Forbes, of St. Louis, addressed the Society relative to the formation of a State Society in Illinois. He stated that one had been formed in Michigan, and he had understood that some initiative steps had been taken in Chicago looking to the organization of a State Society for Illinois, and wished to know if that was the case, adding that in the opinion of the Dentists of St. Louis, owing to the peculiar position in which the State of Missouri is now placed, any attempts to effect a State organization in that State would be unadvisable. He, however, thought the time had come when the interests of the profession in Illinois would be forwarded by a State organization.

Dr. J. P. Abell, of Chicago, replied that he had not been a regular attendant at the meetings of the Chicago Society, but was not aware that any movement of the kind had been made.

Dr. Forbes said that he wished to make a few remarks on another subject, one which had been often discussed, and one of great interest to dentists. He referred to the question of the presence of organized nervous tissue in the tubuli of dentine

Since his arrival in the city he has been shown some fine microscopic preparations, which he thought would go far towards settling this long disputed question. The specimens belonged to Dr. W. W. Allport, of Chicago; and considering the subject a most important one, he would move the appointment of a committee of three to examine the specimens in Dr. Allport's possession, and report to a future meeting of the Society.

The motion was carried, and the Chair appointed Drs. Forbes, Spalding, and Abell as the committee.

The Society then proceeded to the election of officers for the ensuing year with the following result:

For President, J. P. Abell, of Chicago; First Vice-President, Joseph Mansfield, of Michigan; Second Vice President, W. H. Eames, of St. Louis; Recording Secretary and Treasurer, C. W. Spalding, of St. Louis; Corresponding Secretary, E. A. Bogue, of Chicago. Executive Committee, W. W. Allport, J. Ward Ellis, H. E. Peebles, W. N. Morrison, and J. C. Fuller.

The Society then adjourned till 3 o'clock, P. M.

AFTERNOON SESSION.

The Society re-assembled at 3 o'clock, pursuant to adjournment, Dr. Peebles, the retiring President, in the Chair.

Dr. Allport moved that the Society proceed to the election of delegates to the American Dental Association, which is to meet at Niagara Falls, on the 26th inst. Carried. Whereupon the following delegates were chosen: Drs. H. E. Peebles, I. Forbes, A. M. Leslie, and C. W. Spalding, of St. Louis; Drs. W. W. Allport, L. P. Haskell, and J. P. Abell, of Chicago; and Dr. D. W. Perkins, of Milwaukee.

At the request of the President, Drs. Allport and Mansfield conducted the President elect to the chair.

The President on taking the chair, said that his feelings were of a peculiar character when he considered the circumstances and the unexpected manner of introducing him into the place he now occupied. It had been once his fortune to reside in an eastern State,—an extremely eastern State. He remembered, while he lived there, that a certain wealthy ship owner was in the practice of introducing his sons as captains of his vessels before they had sufficient experience to command them. In such cases the sailors always said that such captains came in through the cabin window. He had so little intercourse with the members of the Society that he might almost be said to have come into the chair through the cabin window. But, whatever might be the circumstances, he felt bound to serve the Society to the best of his ability. He remembered once to have read of an ancient monarch who, while conducting his victorious armies from one country to another, and when about entering the new country, would turn round and, with the greatest solemnity, take his leave of the deities who were supposed to preside over the land. He would turn towards the new country and, in the most approved method, commend himself to the divinities of the land he was about to enter.

The President hoped they would allow him to take his leave of the past and to most heartily and cheerfully to forget it, and he gave his assurance that he would to the utmost of his ability serve the interests of the Society of which he had been elected President. He believed that it had almost become an universal maxim that the estimate an individual placed upon himself would be accepted by the public. He believed that this was the case in regard to organized bodies like the Dental Society. It seemed to him, then, that according to the estimate they placed on themselves, in that position would the public receive them.

He remembered the time when a dentist was considered little more than a mountebank. He could pull teeth and file teeth, but in the best possible light they could look upon him he seemed nothing but a live mountebank. The profession had vastly improved, and if they were only true to themselves, he thought they could not exalt themselves too highly. As they regarded the profession, so would the public regard it. He believed there was no profession more honorable or more useful. He knew of none that was more so. He hoped, therefore, they would all devote themselves to the advancement and interests of the Dental Profession, and he would promise to serve the society to the utmost of his power.

The Treasurer's report showed a balance of $132 69 on hand.

The report was audited by the Ex-Committee.

Dr. Allport moved that $100 be appropriated for the instruction of some deserving young man at a dental college. He said he made the motion from the fact that they had an overplus in the treasury which they did not require to use at present. He did not think they could put it to a better use than providing for the tuition of a deserving young man to be selected by a committee of three to be named by the chair.

The motion was discussed by Drs. Allport, Ellis, Spalding, and Mansfield.

The motion was carried and the chair appointed on the committee Drs. Allport, Ellis, and Spalding.

Dr. Spalding made a verbal report as delegate to the American Dental Association last year. He wished to be excused from making a written report as he felt he could not do justice to the occasion, after having seen the published transactions of the association.

Dr. Allport moved that the request be granted. Carried.

Dr. Allport who had brought a powerful microscope and some very valuable dental preparations into the room, said that it had been a matter of much discussion, not only in the journals but in the books issued on Dentistry, as to what the body of the tooth was composed of, and what caused the sensitiveness when it was being cut. In the structure of the tooth, as they were all aware, there were certain tubes running in a parallel and horizontal direction, visible only under a powerful microscope. Kollker, Lunt and Leidy had contended that these tubes were filled with fluid, and that the sensitiveness was caused simply by pressing on the ends of these tubes and forcing the fluid back. The condition of the dentine was such as led him to believe, after many experiments, that what he had referred to were absolute tissues and not tubes filled with nerve fluid. Tomes, of London, had published a book on the subject, with illustrations, to show that these tubes were filled with nerve fluid. If this conclusion was correct, that these tubes were nervous fibres, then it would enable them to settle a matter long under discussion. Tomes had explained the manner in which these preparations had been made, and about two years ago he (Dr. Allport) had made several preparations on the same plan, with the view of satisfying himself as to the correctness of the hypothesis advanced by Tomes. He had submitted them to Dr. Leidy and Dr. McQuillan, of Philadelphia. They did not agree with him, but he still adhered to the opinion that the tubes contained colorless fluid, and that the acids used in making the preparations coagulated the fluid which enabled it to be easily drawn from the tube. He had made preparations without using the acid, and in his opinion, the contents of the tubes were a tissue.

The members were invited to examine the specimens for themselves.

Dr. Spalding suggested that the next meeting of the Society be held during winter. Michigan, Indiana, and Iowa have all established State societies, and other members were now included to the States of Wisconsin, Illinois and Missouri. He thought they might select St. Louis as the place in which to hold the winter meeting if they agreed to hold one. Complaint had often been made when St. Louis was proposed for the summer meeting that it was too hot to go there.

Dr. Forbes opposed a meeting in winter.

Dr. Allport moved that the next meeting be held on the third Wednesday of January, 1865, and that it be considered the Ninth Annual Meeting. Carried.

Dr. Ellis moved that the meeting be held in St. Louis. Carried.

Dr. Spalding moved a cordial vote of thanks to the proprietors of the Tremont House for their courtesy in providing a commodious room for the meetings of the Society. Carried unanimously.

A vote of thanks having been tendered to the retiring officers, the Society adjourned to meet in St. Louis on the third Wednesday (the 18th) of January, 1865.

